# SPECTRA: An Integrated Knowledge Base for Comparing Tissue and Tumor-Specific PPI Networks in Human

**DOI:** 10.3389/fbioe.2015.00058

**Published:** 2015-05-08

**Authors:** Giovanni Micale, Alfredo Ferro, Alfredo Pulvirenti, Rosalba Giugno

**Affiliations:** ^1^Department of Computer Science, University of Pisa, Pisa, Italy; ^2^Department of Clinical and Molecular Biomedicine, University of Catania, Catania, Italy

**Keywords:** tissue, tumor, database, proteins, interactions

## Abstract

Protein–protein interaction (PPI) networks available in public repositories usually represent relationships between proteins within the cell. They ignore the specific set of tissues or tumors where the interactions take place. Indeed, proteins can form tissue-selective complexes, while they remain inactive in other tissues. For these reasons, a great attention has been recently paid to tissue-specific PPI networks, in which nodes are proteins of the global PPI network whose corresponding genes are preferentially expressed in specific tissues. In this paper, we present SPECTRA, a knowledge base to build and compare tissue or tumor-specific PPI networks. SPECTRA integrates gene expression and protein interaction data from the most authoritative online repositories. We also provide tools for visualizing and comparing such networks, in order to identify the expression and interaction changes of proteins across tissues, or between the normal and pathological states of the same tissue. SPECTRA is available as a web server at http://alpha.dmi.unict.it/spectra.

## 1 Introduction

In the last 10 years, there has been a rapid growth of available protein–protein interaction (PPI) data. They represent all known physical interactions between proteins within a cell. The collection of PPI data yields a network depicting a global overview of the relationships between proteins.

Nowadays, PPIs of species and associated data are stored in many databases, which usually are weekly or monthly updated. Primary sources of PPI data include BioGRID (Stark et al., 2006), DIP (Xenarios et al., 2000), HPRD (Peri et al., 2004), IntAct teorchard2013mintact, and MINT (Licata et al., 2012).

DIP (Xenarios et al., 2000) was the first database, which combined information from multiple observations and experimental techniques into networks of interacting proteins for different species. HPRD (Peri et al., 2004) contains manually curated proteomic information regarding human proteins, which are annotated and linked to OMIM database (Hamosh et al., 2005). BioGRID (Stark et al., 2006) collects protein–protein and genetic interactions for all major model organisms trying to remove redundancy and create a single mapping of interactions. The IntAct database (Orchard et al., 2013) provides tools for both textual and graphical representations of protein interactions. Interacting proteins can be annotated with GO terms for functional analysis. MINT (Licata et al., 2012), which is based on the IntAct database infrastructure, collects experimentally verified PPIs by extracting experimental evidences from the scientific literature.

Some databases integrate PPIs data of human and other organisms from primary sources, by removing redundancies and assigning a unique reliability score. These include STRING (Franceschini et al., 2013), IRefIndex (Razick et al., 2008), ConsensusPathDB (Kamburov et al., 2013), and HitPredict (Patil et al., 2011).

STRING (Franceschini et al., 2013) combines physical interaction data and curated pathways of different organisms with predicted interactions from text mining, genomic features and interactions transferred from model organisms based on orthology. IRefIndex (Razick et al., 2008) is a set of tools to index and retrieve proteins and interactions from major public databases. Indexes are built according to protein sequences and taxonomy identifiers and mapping scores evaluate the quality of the mapping. ConsensusPathDB (Kamburov et al., 2013) integrates human protein–protein interactions, biochemical pathways, gene regulatory, and drug–target interactions into a global network, containing genes, proteins, and metabolites, which can be visualized, analyzed, and annotated. HitPredict (Patil et al., 2011) combines PPI data from IntAct (Orchard et al., 2013), BIOGRID (Stark et al., 2006), and HPRD (Peri et al., 2004), by assigning a confidence score based on sequence, structure, and functional annotations of the interacting proteins. The reliability score is calculated using the Bayesian networks.

The analysis of PPI networks has provided novel biological insights on the function of many previously uncharacterized proteins in *Human* through module identification (Bader and Hogue, 2003; Adamcsek et al., 2006; Mete et al., 2008; Rhrissorrakrai and Gunsalus, 2011), network querying (Ferro et al., 2007; Banks et al., 2008; Bruckner et al., 2010), and network alignment (Flannick et al., 2006; Kalaev et al., 2009; Liao et al., 2009; Sahraeian and Yoon, 2013; Micale et al., 2014a) algorithms. Furthermore, the annotation of PPI networks with external data (i.e., diseases, expression data, phenotypes) has helped to classify genes according to the expression profiles (Dao et al., 2011), predict new gene–disease associations (Huang et al., 2012; Zhao et al., 2012), and discover new drugs (Huang et al., 2012; Alaimo et al., 2013; Csermerly et al., 2013).

These tasks have been accomplished thanks to the availability of authoritative repositories of gene expression data in normal/cancer tissues and at different diseases stages (Uhlen et al., 2010; Barrett et al., 2013; Rustici et al., 2013). For example, ArrayExpress (Rustici et al., 2013) and GEO (Barrett et al., 2013) include gene expression data from microarray and high-throughput sequencing experiments, which can be easily queried or downloaded. Users can also submit data directly by using the standard MIAME format. More recently, new projects have started with the aim of cataloging tissue or tumor sequencing data. The Cancer Genome Atlas (TCGA)^1^ collects complete high-throughput genome data (clinical information, expressions data, methylations, mutations) for specific cancer tissues, with the purpose of helping the diagnosis and the treatment of cancers. The Human Protein Atlas (Uhlen et al., 2010) is a database with histological images showing the spatial distribution of proteins in normal and cancer tissues. Protein Atlas contains also transcription expression levels, protein expression profiles, and subcellular localization data.

All above PPI networks data are constructed by ignoring the role of proteins in human tissues. On the other hand, human diseases often occur in specific tissues (Lage et al., 2008). Some genes can be predominantly expressed in one or few tissues and can control the formation of protein complexes (Emig and Albrecht, 2011). Furthermore, genes can use alternative splicing as a powerful mechanism to enlarge the number of their interactors and perform distinct functions in different tissues (Emig and Albrecht, 2011). Therefore, the integration of PPI networks with tissue-specific gene expression data can help to highlight the role of some genes in specific disease or tumors. The result of such integration gives the so called Tissue-Specific PPI (TS-PPI) network (Bossi and Lehner, 2009), which is a subgraph of a PPI network where the genes corresponding to both interacting proteins are expressed in one or more selected tissues.

Some studies focus on the analysis of global and local properties of TS-PPI networks. In Bossi and Lehner (2009), authors prove that most housekeeping proteins form highly tissue-specific protein interactions, suggesting a key role of those proteins in tissue-specific biological processes. Emig and Albrecht (2011) show that the number of tissue-specific proteins is very low and the receptor-activated signaling processes and the transcriptional regulation are two key factors for tissue specificity. In Souiai et al. (2011), a gradient model is used to describe the structure of TS-PPI networks, containing interactions of regulatory and developmental functions at the core of the TS-PPI network and physiological functions at the periphery.

Several recent works highlight the advantages of using TS-PPI networks. In Lopes et al. (2011), a set of proteins related to the response of viral infection in a TS-PPI network lead to a more reliable functional enrichment. Magger et al. (2012) use TS-PPI networks to improve the prioritization of candidate disease-causing genes with respect to a generic PPI network. In Chen and Wang (2012), authors identify functional modules in TS-PPI networks using CFinder (Adamcsek et al., 2006) and show that they exhibit more biological meaning than modules in a PPI network. Xiao et al. (2014) propose a new method for the identification of multi-tissue gene co-expression networks associated with specific functional processes relevant for phenotype variation and disease in humans. Barshir et al. (2014) show that genes causing hereditary diseases tend to have higher transcript levels and more interacting partners in the TS-PPI network of disease tissues than in the TS-PPI network of unaffected tissues.

To the best of our knowledge, few tools are available for querying and analyzing TS-PPI networks (Barshir et al., 2013; Nersisyan et al., 2014). CyKeggParser (Nersisyan et al., 2014) is a Cytoscape app for generating and analyzing tissue-specific KEGG pathways. Pathways can be checked for inconsistencies and modified based on gene expression data from normal and cancer tissues. TissueNet (Barshir et al., 2013) is a dataset of TS-PPIs in humans, which integrates a collection of four PPInetworks (BioGRID, DIP, IntAct, and MINT) with three expression datasets (GEO, Human Protein Atlas, and Illumina Body Map 2.0). The database provides a web interface for retrieving tissue-specific interactions of a query protein. However, it handles only 16 normal tissues and does not provide any tool for the analyses of TS-PPI networks.

In this paper, we propose SPECTRA (SPECific Tissue/Tumor Related PPI networks Analyzer), a framework to build and analyze TS-PPI networks. SPECTRA integrates tissue and tumor-specific gene expression data from the most authoritative online repositories such as Protein Atlas, ArrayExpress, GEO, and TCGA. Expression data are then integrated with high-quality protein–protein interactions, taken from HPRD, BioGRID, MIPS, IntAct, and the work of Havugimana et al. (2012). We provide a web interface for constructing, visualizing, and comparing TS-PPI networks, with the aim of identifying differential interaction/expression patterns in TS-PPI networks (i.e., distinct tissues, or normal and pathological states of the same tissue). The TS-PPI networks together with the results of differential analysis can be easily visualized by using Cytoscape facilities (Shannon et al., 2003) and downloaded as text files for further investigations. SPECTRA is free for all users and available at http://alpha.dmi.unict.it/spectra.

## 2 Materials and Methods

SPECTRA combines protein–protein interactions in human with gene expressions, by integrating 13 authoritative resources. The final integrated SPECTRA database contains 16,435 protein coding genes and 175,841 gene interactions (GIs), 1,350,637 tissue-specific gene expression data entries covering 107 normal tissues, and 2,171,808 tumor-specific expression data entries covering 160 different tumors.

### 2.1 Interaction datasets

Human protein interaction data were taken from BioGRID^2^, DIP^3^, a recent work by Havugimana et al. (2012), HPRD^4^, IntAct^5^, and MINT^6^.

Table 1 describes the features of the PPI networks integrated in SPECTRA. Networks taken from the work of Havugimana et al. (2012), IntAct and MINT are weighted with edge weights ranging in [0,1], while the other PPI networks are unweighted. Proteins of the considered PPI networks, including splicing isoforms, were first mapped to the corresponding gene. Next, a global GI network was built, by collecting all interactions reported in at least one dataset. We assigned to each edge a pair consisting of the average value of weights across the datasets that report that interaction and the percentage of datasets giving the interaction (dataset coverage). Average edge weights range from 0.131 to 1.

**Table 1:** **Features of PPI networks integrated in SPECTRA**.

Network	Nodes	Edges	Type
BioGRID	15,290	135,677	Unweighted
DIP	2,338	3,427	Unweighted
Havugimana et al. (2012)	3,003	13,989	Weighted
HPRD	9,506	37,054	Unweighted
IntAct	11,637	63,030	Weighted
MINT	6,551	18,478	Weighted

**Figure 1:**
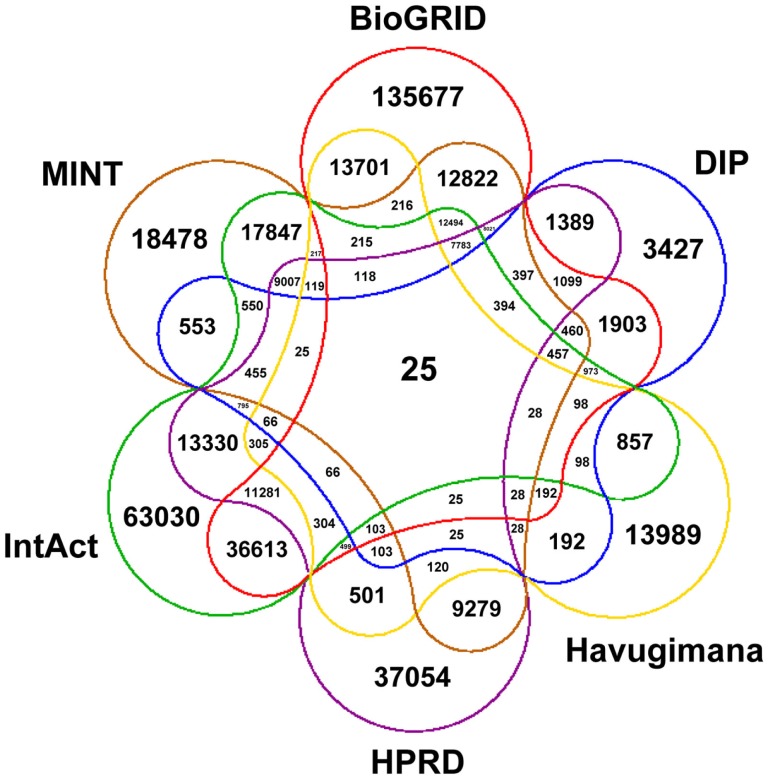
**Venn diagram showing the number of common interactions across PPI datasets in SPECTRA**.

**Figure 2:**
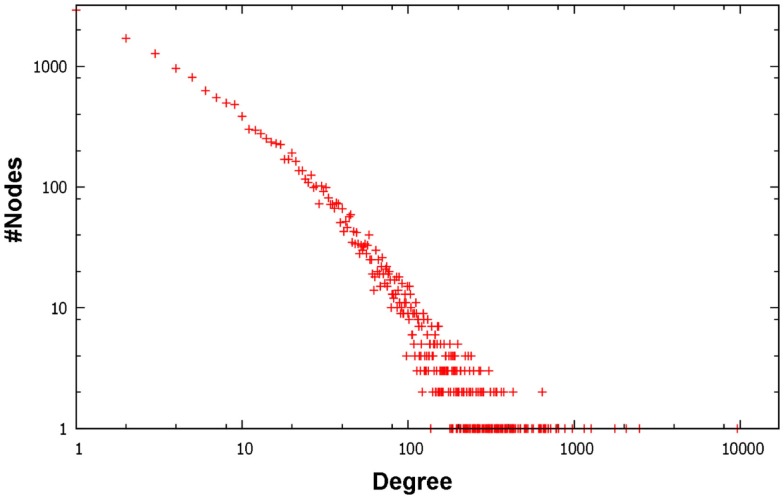
**Log–log plot of degree distribution of the final integrated PPI network in SPECTRA**.

Figure 1 depicts a Venn diagram of common gene interactions between PPI datasets. Interaction databases generally show low overlap, with only 25 interactions shared by all datasets and only 7,783 interactions in common between MINT, BioGRID, IntAct, and HPRD, which are the biggest ones. The final integrated network has 16,435 nodes, 175,841 edges and 17 connected components, with a high average diameter (9) and low clustering coefficient (0.289). The average degree is 21.398 and the degree distribution follows a power law (Figure 2).

### 2.2 Expression datasets

Gene expression data for various tissues and tumors were downloaded from ArrayExpress^7^, GEO^8^, ProteinAtlas^9^, and TCGA (see text footnote 1). Table 2 lists the gene expression datasets integrated within SPECTRA, the platform used to detect the expressions and the number of covered tissues and tumors.

**Table 2:** **Features of expression datasets integrated in SPECTRA**.

Dataset	Platform	Tissues	Tumors
E-MTAB-62 (Lukk et al., 2010)	GPL96	46	110
GDS181 (Su et al., 2002)	GPL91	29	6
GDS596 (Su et al., 2004)	GPL96	57	5
GDS1096 (Ge et al., 2005)	GPL96	36	0
GDS3113 (Dezso et al., 2008)	GPL2986	32	0
ProteinAtlas	GPL11154	28	33
TCGA	Agilent G4502A-07-3	0	27

**Figure 3:**
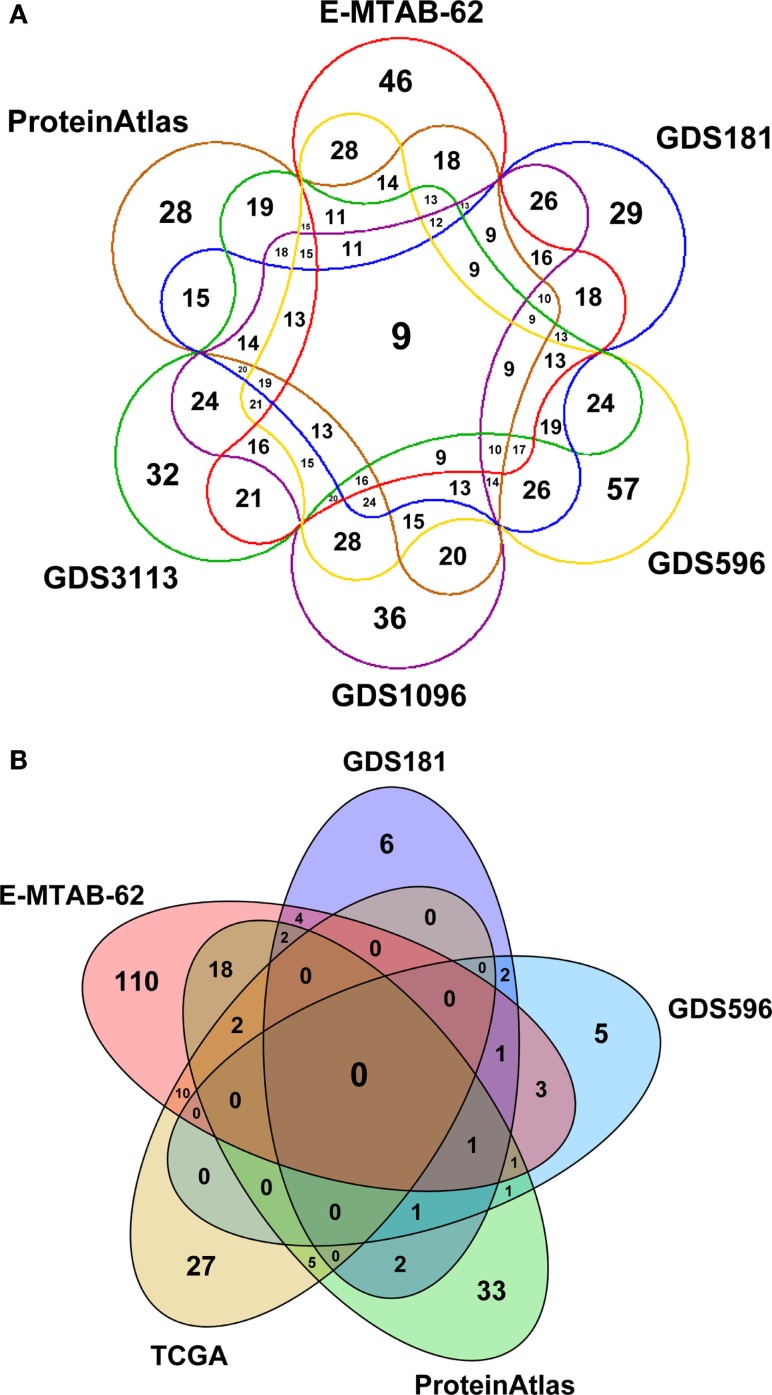
**Venn diagrams depicting: (A) the number of common tissues and (B) the number of common cancer types across expression datasets in SPECTRA**. The zero overlap among the cancer datasets is due to the fact that cancers are considered according to their specific names and not to their class (breast cancer, prostate cancer, etc.).

Figure 3 depicts a Venn diagram of common tissues and tumors across expression datasets. While tissue names are generally shared, tumor names are much differentiated, resulting in a poor overlap between datasets. In particular, TCGA contains data for very specific tumors and partially overlap only with E-MTAB-62 dataset, which is the richest one. Note that the numbers reported in Figure 3 only refer to specific tumors and not to tumor classes. So, for instance, “breast carcinoma” and “breast adenocarcinoma” are considered distinct tumors, even though they belong to the same class of tumors, “breast cancer.”

As regards the integration of expression data, we followed the work of Guo et al. (2013), where authors show that there is a positive correlation between normalized Affymetrix and RNA-Seq data. We performed RMA normalization (McCall et al., 2010) for datasets based on Affymetrix platforms (GDS181, GDS596, and GDS1096), using the corresponding R Bioconductor package. For GDS3113, ProteinAtlas, and TCGA, we first computed the log_2_ of the number of fragments and then we normalized values using the quantile normalization method of Bolstad et al. (2003). For GDS181, GDS596, and GDS1096 datasets, normalized values were computed from raw data. Then, probes, which were present in a particular microarray dataset, were mapped to the corresponding genes. Finally, the expression of a gene for a specific tissue was computed as the average expression value of probes mapping to that gene in the tissue. For GDS3113, ProteinAtlas, and TCGA, instead, we directly normalized gene expression values for different tissues. We did not normalize EMTAB-62 data, since its source values were already normalized with RMA.

Finally, we assigned to each pair gene–tissue a unique positive expression score, given by the average normalized expression value of the gene in that tissue, according to the different datasets. Expression scores in SPECTRA range from 3.566 to 17.366 for tissues and from 0.01 to 17.343 for tumors.

### 2.3 Data schema

SPECTRA database is structured as a MySQL relational database with six tables: *Genes*, *Tissues*, *Tumors*, *Interactions*, *Expr_normal*, and *Expr_tumor*.

The *Genes* table contains the list of all expressed and interacting genes. Each entry is identified by the gene symbol and contains associated data, including a description string, aliases, and cross references to Entrez Gene (if available).

The *Tissues* and *Tumors* tables have the same structure. Tissues and tumors are associated to different classes, depending on the organism part they refer to. Each entry is identified by a unique number and contains a description and the corresponding class. SPECTRA contains 26 distinct classes of tissues and 32 distinct classes of tumors.

The *Interactions* table lists all the PPIs integrated in SPECTRA. Interactions are identified by a couple of gene symbols and the edge weight for each integrated dataset (when available) is stored, together with the average interaction weight across dataset reporting that interaction and the dataset coverage.

*Expr_normal* and *Expr_tumor* contain all the gene expressions in normal and cancer tissues. The unique identifier of *Expr_normal* is a couple gene–tissue, while entries in *Expr_tumor* are uniquely identified by the couple gene–tumor. In both tables, the normalized expression value for each integrated dataset (where available) and the average expression score are included as associated data.

### 2.4 An algorithm for differential local alignment of TS-PPI networks

TS-PPI networks are compared in SPECTRA for identifying patterns of differential gene expressions between multiple TS-PPI networks.

Our goal is to find conserved sub-regions in the TS-PPI networks, which maximize the difference of expression values of aligned genes. The problem is related to that of finding maximal-scoring connected subgraphs, which is NP-hard, even in a common simpler setting where the aligning TS-PPI networks have the same set of nodes and edges (e.g., TS-PPI networks built starting from different expression data and the same interaction datasets) (Ideker et al., 2002).

In the case of two TS-PPI networks with the same set of nodes and edges (representing for instance case and control expression data), heuristic (Ideker et al., 2002; Sohler et al., 2004; Cabusora et al., 2005; Rajagopalan and Agarwal, 2005; Guo et al., 2007) and exact (Dittrich et al., 2008) solutions have been proposed. However, as far as we are concerned, no solutions are known for the multiple case. Here, we propose an approximate solution to the multiple differential alignment problem based on a modified version of the GASOLINE algorithm (Micale et al., 2014a). For simplicity, we consider TS-PPI networks with no multiple edges between two nodes.

#### 2.4.1 The GASOLINE Algorithm

**Figure 4:**
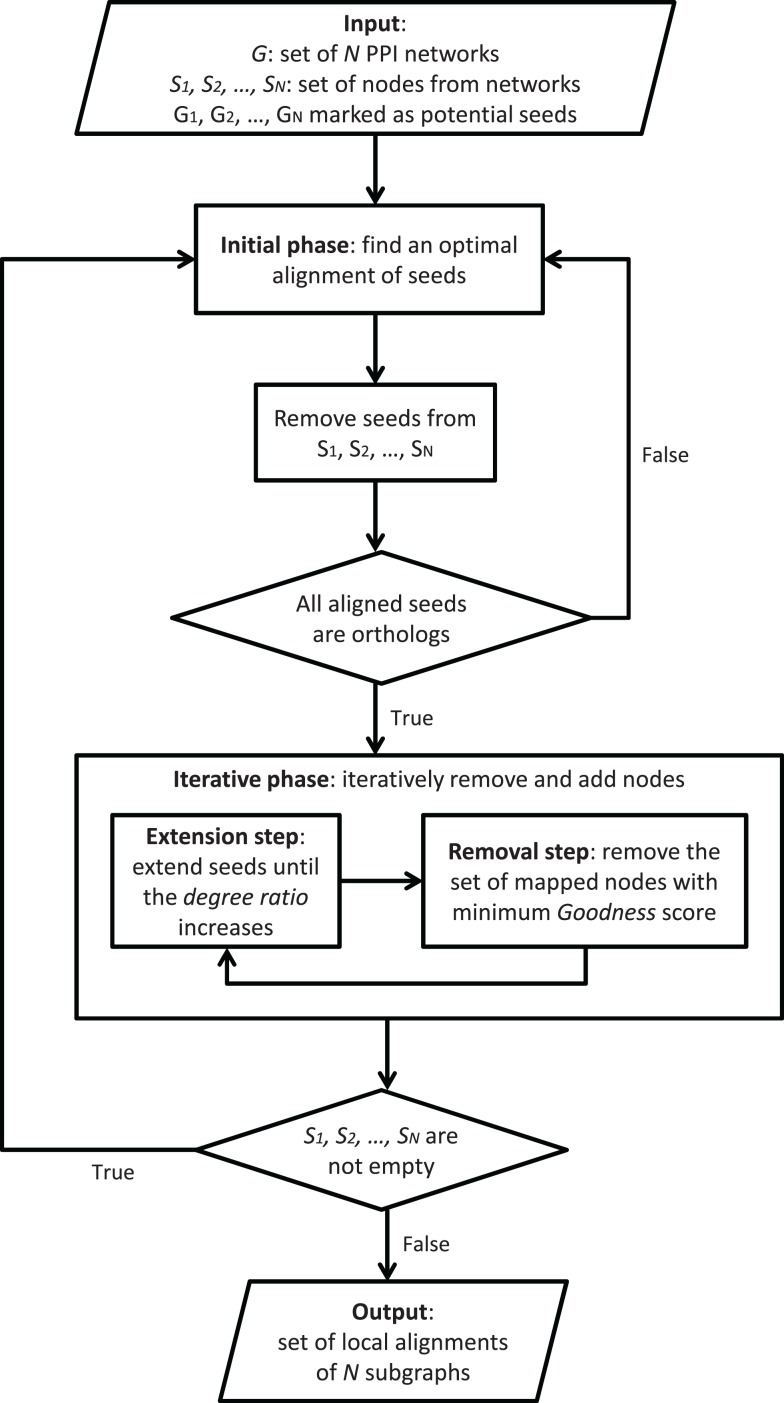
**General description of GASOLINE**.

GASOLINE (Micale et al., 2014a) is a greedy and stochastic algorithm for multiple local alignment of protein–protein interaction networks. Flowchart in Figure 4 provides a general description of GASOLINE.

Given *N* weighted PPI networks of different species, where edge weights are probabilities of interaction between proteins, local alignment aims at finding a set of connected subnetworks, one from each network, that are conserved in their sequence and interaction pattern. Such subnetworks could represent evolutionary conserved complexes or pathways across different organisms.

Such a problem is related to subgraph isomorphism, which is known to be NP-complete (Cook, 1971). GASOLINE proposes an approximate solution through a stochastic-greedy strategy consisting of two phases.

In the first step, called bootstrap phase, we look for orthologous proteins across the networks and build a set of seeds. The set of seeds initially consists of proteins, one from each network, and includes all the starting nodes of the suboptimal local network alignment.

The second step, called iterative phase, repeatedly adds (extension step) or removes (removal step) nodes in the network alignment, trying to maximize the final alignment score. Each extension step adds, in each network, a single node to the corresponding seed. During the extension step, the seeds grow up producing a set of subgraphs, one from each network. The extension process is regulated by a properly defined degree ratio measuring the average density of the aligned subgraphs with respect to their neighbors in the networks. The extension is performed until the degree ratio increases.

Each removal step replaces from the current alignment the set of proteins (one from each network) with minimum topology similarity score.

The bootstrap phase and each extension step are performed through Gibbs sampling (Geman and Geman, 1984). In both cases, the Gibbs sampling builds a chain, where each state represents a combination (i.e., alignment) of single proteins, one from each network. First, a random initial state is selected. Then, the sampling method iteratively performs a transition from a state to another, by replacing a randomly chosen protein of the current alignment with a protein of the same network, according to a properly defined transition probability distribution.

Due to its non-deterministic nature, different iterations of GASOLINE may produce different local alignments. The above steps are iterated to produce a set of local networks alignments, which are then ranked according to an Index of Structural Conservation (ISC) score. ISC score measures the percentage of conserved interactions in the final alignment. The higher is ISC, the better is the alignment.

GASOLINE implements preprocessing and post-processing steps. During preprocessing, the search space for potential seeds is reduced. This is obtained by marking only proteins having orthologs in all aligning networks and with a significant interaction degree in each network. All marked nodes in each network *G_i_*(1 ≤ *i* ≤ *N*) are added to a set called *S_i_*. These sets will be used in the initial phase and will be updated at each iteration. Finally, a post-processing filters the final set of local alignments returned by GASOLINE by removing highly overlapping complexes.

GASOLINE does not allow many-to-many mapping between aligned nodes. However, experimental results show that the algorithm can produce more reliable results than methods implementing many-to-many mapping. Moreover, GASOLINE is clearly faster than the state-of-art algorithms (Micale et al., 2014a).

#### 2.4.2 The Adapted GASOLINE

We implemented a customized version of GASOLINE to compare two or more Tissue-Specific PPI (TS-PPI) networks for local differential alignment problem. GASOLINE algorithm was extended to deal with gene expressions as weights to the nodes.

Let *A* and *B* two genes and *Expr(A)* and *Expr(B)* their expression values, with *Expr(A) ≥ Expr(B)*. In order to evaluate the expression difference between *A* and *B*, we compute the *log fold change*, defined as follows:

(1) $$\text{LogFold}\left(A,B\right)={\text{log}}_{2}\left(\frac{\mathrm{Expr}\left(A\right)}{\mathrm{Expr}\left(B\right)}\right)$$

Given *N* TS-PPI networks and a set of aligned genes *G* = {*G*_1_, *G*_2_, … , *G_N_*}, one for each TS-PPI network, MaxLogFold is the maximum value of LogFold function among all pairs of genes in *G*:

(2) $$\text{MaxLogFold}\left(G\right)=\text{max}\left\{\text{LogFold}\left(G_{i},G_{j}\right)\forall 1\leq i<j\leq n\right\}$$

We applied the following changes to original GASOLINE algorithm:

- We included the LogFold function in the Gibbs sampling procedure of bootstrap and iterative phases, by multiplying it by the topology and homology scores in the computation of node similarities;
- The number of iterations of Gibbs sampling both in the bootstrap and in the extension phase is governed by a new parameter, is *α*, which is a probability threshold related to *N*, the number of networks, according to the following formula:
  (3) $$k=\text{max}\left\{k\prime :{\left(\frac{N-1}{N}\right)}^{k\prime }>\alpha \right\}$$
  where $P={\left(\frac{N-1}{N}\right)}^{k\prime }$ is the probability that a gene is never selected in *k′* consecutive iterations of Gibbs sampling. The idea is to stop Gibbs sampling when an alignment does not change for *k* consecutive iterations. The lower is *α*, the higher is *k*, so the more precise and slower will be the sampling procedure:
- We introduced a new threshold, *MaxLogFoldThreshold*, for the value of MaxLogFol function, and we used it to tune the extension process in place of the degree ratio: in particular, we extend the current alignment until the average value of *MaxLogFold* between the sets of aligned nodes is above such a threshold;
- In the remove phase, the set of aligning nodes with minimum value of MaxLogFold is deleted from the current local alignment;
- Given a local alignment *A* = {*A*_1_, *A*_2_, … , *A_w_*}, where *w* is the size of the alignment and *A*_1_, … , *A_w_* are the set of aligned genes, an average value of MaxLogFold(*A_i_*) is computed together with the *ISC* score to evaluate the quality of the alignment.

## 3 Results

SPECTRA is a framework for retrieving and analyzing protein–protein interaction data specific for a given set of normal or cancer tissues. The underlying graph model in SPECTRA is the Tissues-Specific PPI network (or TS-PPI network), in which the genes of corresponding interacting proteins are both expressed in one or more tissues. The architecture of SPECTRA is composed by (i) the *searching tool*, which allows to build TS-PPIs; (ii) the *comparison tool* to look for shared differential expressions patterns between genes of two or more TS-PPI networks. Results can be graphically visualized by using Cytoscape.js or downloaded as text files.

### 3.1 SPECTRA search tool: Building TS-PPI networks in SPECTRA

**Figure 5:**
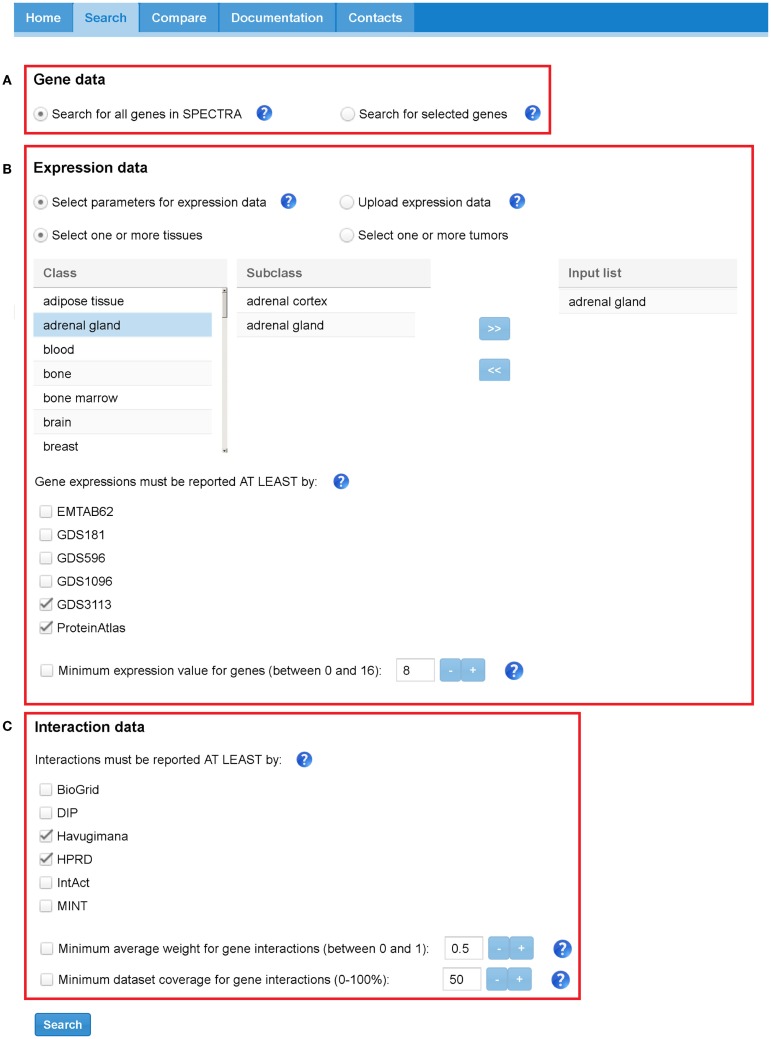
**SPECTRA search tabbed panel**. Red boxes highlight the three sections: **(A)** “Gene data,” **(B)** “Expression data,” and **(C)** “Interaction data.” In this case, the parameters have been set to indicate that we want to retrieve all the interactions that are present at least in Havugimana and HPRD, involving genes that are expressed in adrenal gland” tissue according at least to GDS3113 and ProteinAtlas. In this example, we neither restrict our search to a predefined set of genes nor provide a threshold for interaction weights, dataset coverage, and expression scores.

SPECTRA builds TS-PPI networks starting from a user-defined set of genes, tissues, expression data, and interaction data. Figure 5 depicts the search interface of SPECTRA.

In the “Gene data” section (Figure 5A), the user can look for all genes expressed in a set of tissues or restrict the search to a specific list of genes. Genes can be provided with their official names or Aliases (e.g., Ensembl Gene, Entrez Gene, Affy).

In the “Expression data” section (Figure 5B), the user limits the search to a set of tissues/tumors and to a set of expression datasets or uploads a text file with custom expression data. Note that the two options are mutually exclusive, that is, all the settings concerning datasets and tissue/tumors will be ignored if the user provides a custom text file. Available tissues and tumors in SPECTRA are listed in a table and can be easily included in the input query list with a double click in each entry. When no data are provided, all the tissues and tumors in SPECTRA are considered. Tissues and tumors are also mutually exclusive, meaning that a TS-PPI network built-in SPECTRA cannot contains interactions defined on both normal and tumor tissues. However, two TS-PPI networks defined upon a specific set of tissues and tumors, respectively, can be always compared for differential analysis with the adapted GASOLINE. The user can also select one or more datasets from which the expression have to be reported. When the expression is in other datasets it will be also given. When no dataset is selected, all expression data in SPECTRA are considered.

Finally, a further filter on genes can be applied by indicating a threshold for the minimum normalized value of gene expressions to be considered.

The “Interaction data” section (Figure 5C) contains the parameters for filtering interaction data. As above, the user can select one or more datasets where protein interactions have to be reported. If no interaction dataset is selected, all PPIs in SPECTRA are considered. A threshold can be provided to select interaction weights above a given value and a minimum dataset coverage.

**Figure 6:**
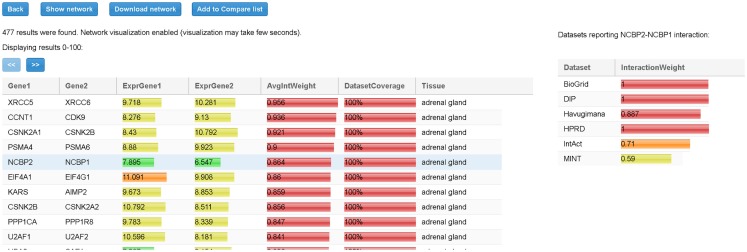
**Result table for the query of Figure 5**. For each interaction in the table we report, the tissue, the average expression scores of interacting genes, and the total interaction weight. Expression scores, weights, and dataset coverage are represented with a colored progress bar (from cyan to red). By selecting a row (in the example the interaction between NCBP2 and NCBP1), detailed data about the interaction are shown to the right. For each dataset, the corresponding interaction weight (when available) is reported (for example, 0.71 for IntAct database).

When all input parameters have been specified, the user clicks on the “Search” button. At the end of the process, all the TS-PPIs found are listed in a result table (Figure 6). For each TS-PPI, we show the interacting genes, the tissues where they are expressed, the expression values of genes in tissues, the average interaction weights and dataset coverages of corresponding proteins. Results are ordered by dataset coverage and average interaction weight. Expression values and interaction weights are depicted with colored progress bar, where colors range from cyan (low values) to red (high values).

**Figure 7:**
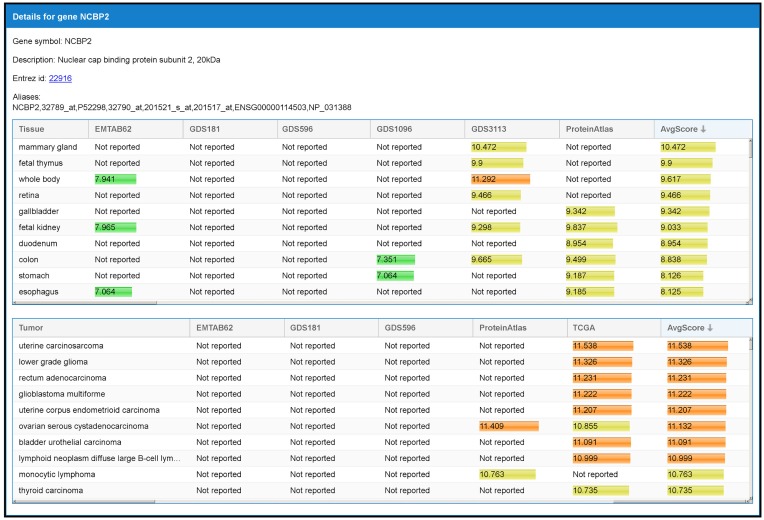
**The panel with detailed information of a gene**. When an interaction is selected from the result table (Figure 6), two panels with additional data, one for each interacting gene, are shown. This example refers to the detailed panel for gene NCBP2, which appears when the row table of Figure 6 is selected. In the detailed panel, the gene symbol, the description, the corresponding ID in Entrez Gene database (when available), and aliases (including references in other databases) are reported. Finally, two tables with the set of tissues and tumors where the gene is expressed are shown. These are shown in decreasing order with respect to the average expression scores.

By selecting a specific TS-PPI in the result table, additional data about the interaction and the interacting genes are shown (Figures 6 and 7). A list of datasets reporting the interaction and the corresponding interaction weight is reported on the right of the result table (Figure 6). Below the result table, two panels with details about the interacting genes are shown (Figure 7). For each gene, description and aliases are provided, together with the lists of tissues and tumors where the gene is expressed, according to the different expression datasets, ordered by expression score.

### 3.2 SPECTRA comparison part: Compare TS-PPI subnetworks

TS-PPI networks can be compared in SPECTRA for identifying patterns of differential gene expressions between multiple TS-PPI networks. The goal is to find conserved sub-regions in the TS-PPI networks, which maximize the difference of expression values of aligned genes.

**Figure 8:**
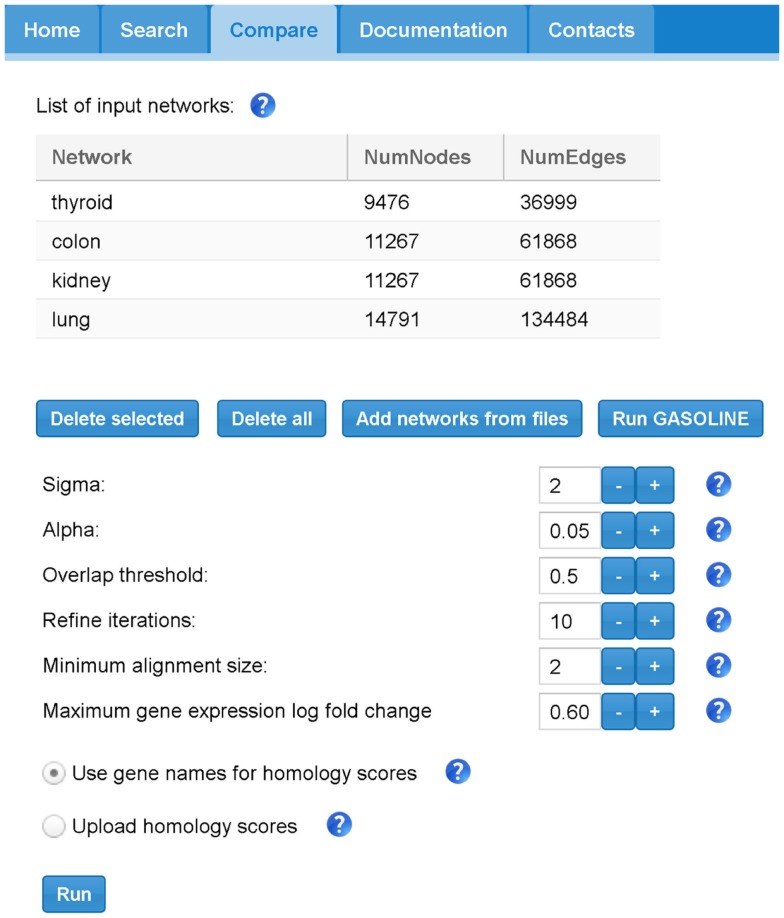
**The SPECTRA compare panel**. In this example, we first loaded 4 different TS-PPI networks from files using the “Add networks” button. Then by clicking on “Run Gasoline” the form for the selection of the adapted GASOLINE input parameters appears.

Figure 8 shows the “Compare” tabbed panel in SPECTRA. Before running the adapted GASOLINE, the user has to upload at least two TS-PPI networks. For each network, the number of nodes and edges are reported. Networks can also be renamed by double clicking on the corresponding cell. Note that uploaded TS-PPI networks with multi-edges between nodes will be always treated as simple networks, where multi-edges are replaced by a single edge with weight equals to the average weight of multi-edges and label given by the concatenation of the multi-edge labels.

Once the networks have been uploaded, the user can click on “Run GASOLINE” button to set the input parameters for the adapted GASOLINE (Figure 8).

We briefly describe their meaning (default values are reported in brackets):

- “Sigma”: the minimum degree of candidate nodes for the initial alignment of seeds (1);
- “Alpha”: a value between 0 and 1, which regulates the number of iterations of Gibbs sampling in the bootstrap and extend phases (default 0.05);
- “Overlap threshold”: a maximum average overlap threshold between local alignments, which is used to remove highly overlapping alignments. It takes values between 0 and 1 (default 0.5, which means 50%);
- “Refine iterations”: the number of iterations of the iterative phase, i.e., extend steps followed by a removal step (default 10);
- “Minimum alignment size”: the minimum size of a local alignment. Local alignments with size lower this minimum size are not reported in final list (default 3);
- “Minimum gene expression log fold change threshold”: value for *MaxLogFoldThreshold*, which controls the extension process (default 0.6).

According to the experiments reported in Micale et al. (2014a,b), we assigned to each parameter default values, which guarantee a good tradeoff between speed and accuracy of GASOLINE.

“Alpha” and “Refine iterations” parameters are strictly related to the stochastic nature of the algorithm. Lower values for “Alpha” and higher values for “Iter Refine” can be assigned to improve accuracy; however, the suggested default values are enough to yield good alignment results. Higher values of “Sigma” can be used to restrict the search to alignments starting from central genes in the input networks and to speedup the algorithm. Lower values of “Overlap threshold” and higher values of “Minimum alignment size” allow to prune the final set of local alignments.

*MaxLogFoldThreshold* is the most critical parameter for GASOLINE. By increasing this threshold, the number and the size of final local alignments can be highly decreased and the algorithm could become much faster. Notice that there is no constant ideal value for *MaxLogFoldThreshold*, because it is highly dependent on the properties of input expression data. For log-transformed gene expression data, like the one which are present in SPECTRA database, low values of *MaxLogFoldThreshold* (0.2–1) are recommended.

Before running the adapted GASOLINE by clicking on “Run GASOLINE” button, the user has to indicate an homology scoring scheme between proteins of different aligning TS-PPI networks (Figure 8). The default naive solution is to use gene names for computing similarities: if two nodes have the same label, then they are considered homologs. Otherwise, user can upload an homology score file.

**Figure 9:**
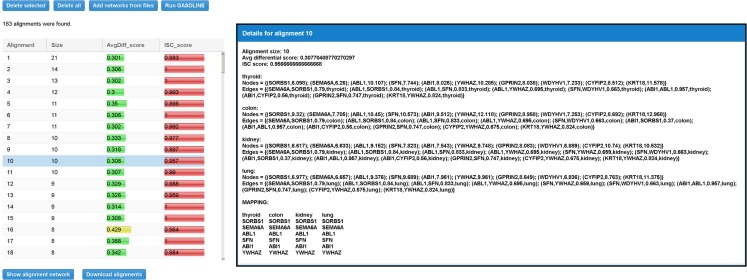
**Result table for the differential local alignment of the four TS-PPI networks of Figure 8 with the Adaptive GASOLINE**. The table reports, for each alignment, the size (i.e., the number of aligned nodes), the average expression difference between aligned nodes, and the ISC (Index of Structural Conservation) score. When the user selects a row in the table, a panel with alignment details is shown to the right. Details include the list of aligned subnetworks (defined by the set of nodes and edges) and the mapping between aligned nodes. Nodes of aligned networks are represented by the corresponding ids, followed by their weights, while edges are represented by the ids of interacting proteins, followed by the interaction weights and the corresponding tissues. Alignment mapping is represented as a matrix where rows contain aligned proteins and columns represent nodes of the same subnetwork.

When the adapted GASOLINE ends, it gives as output a list of local alignments (if any, see Figure 9). For each alignment, the size, the average value of *MaxLogFold*, and the *ISC* score are reported.

By selecting an alignment, its details are reported on the right (Figure 9). Alignment data include the set of nodes and edges attributes. The final mapping of aligned nodes is represented as a matrix in which columns contain nodes of the same network and rows represent the mapped genes.

### 3.3 Alternative input for SPECTRA

User can upload text files in SPECTRA for building and comparing network. Expression data can be provided as text files in the “Expression data” section (Figure 5B) by selecting the Üpload expression data” option. Expression data files should have a matrix format with a row header representing tissues, a column header representing genes, and matrix elements indicating the gene expression value in a tissue.

There are two ways to provide input TS-PPI networks for comparison. User can either upload a text file or create the TS-PPI network with the SPECTRA searching tool and pass it to the comparison page. In the first case, network files are uploaded by clicking on “Add networks from files” in the “Compare” tabbed panel (Figure 8).

TS-PPI network files for comparison follows the same format of the result table in SPECTRA (Figure 6), except for the dataset coverage, with fields separated by tab characters. In the second case, one or more TS-PPI networks for specific tissues are passed to the comparison tool, by clicking on the “Add to compare list” button. The network is then added as input to the comparison list (Figure 8). By default, networks are added with the name of the corresponding tissue, optionally followed by a progressive number whenever two or more TS-PPI networks for the same tissue are already present in the table. Anyway, networks can be later renamed by the user from the comparison table, before running GASOLINE.

In the homology file, needed to run the adapted GASOLINE algorithm, each row contains a pair of nodes of different TS-PPI networks, followed by a positive score value.

### 3.4 SPECTRA output

TS-PPI networks (or subnetworks of them) are downloadable from the result panel, by clicking on “Download network” button (Figure 6). The user can filter the set of tissues upon which the TS-PPI network is defined. TS-PPI networks will be saved into different text files, one for each selected tissue or tumor. The file format is the same of the result table (Figure 6), with fields separated by tab characters.

The set of differential alignments returned by the adapted GASOLINE can be saved as .zip archive. The archive will contain a text file for each alignment. Each file contains the same alignment information reported in Figure 9.

**Figure 10:**
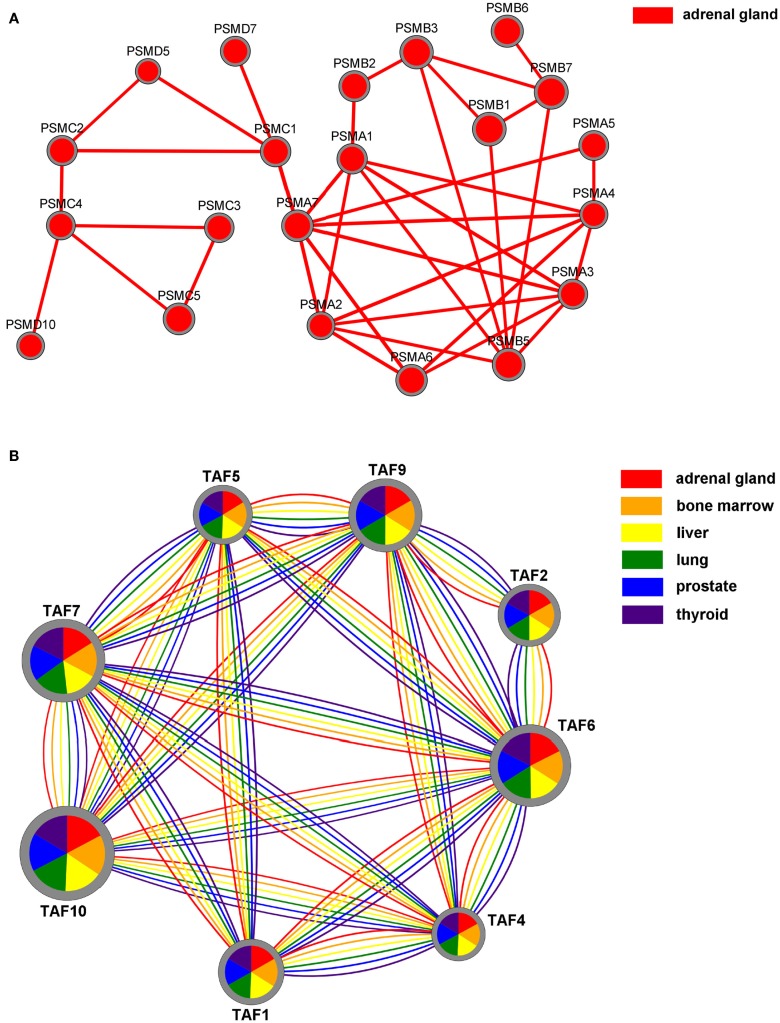
**The Network visualization in SPECTRA**. **(A)** A TS-PPI network for a single tissue; **(B)** A TS-PPI network for multiple tissues. In this case, nodes are represented as pies with slice sizes proportional to the expression of corresponding gene in a tissue. Nodes and edges are colored according to the corresponding tissue and node dimensions are proportional to the total gene expression score.

Results can also be visualized by using Cytoscape.js^10^, a JavaScript library for the analysis and visualization of networks. In the 2D visualization, TS-PPI networks can be navigated and zoomed. A TS-PPI network can be visualized from the result panel (Figure 6). Figure 10 shows two different examples of visualizations of TS-PPI networks within SPECTRA, with one (Figure 10A) or more (Figure 10B) tissues. Nodes and edges are differently colored according to the tissues of the TS-PPI network. Nodes are represented as pies with multiple colored slices. The diameter of the pie is proportional to the total expression score of the gene (considering all tissues of the TS-PPI network) and the size of each pie slice is proportional to the expression score of the gene in the corresponding tissue. Edge line widths are proportional to the interaction weights.

**Figure 11:**
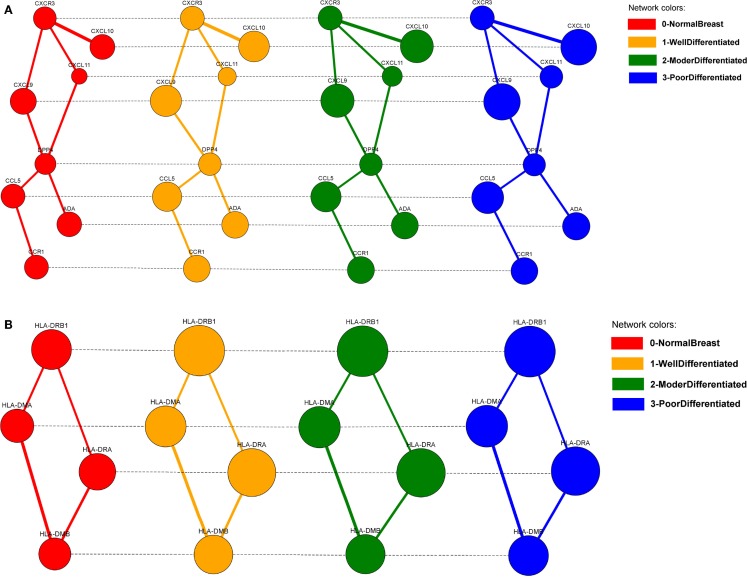
**The two biggest local differential alignments found by the Adaptive GASOLINE for the TS-PPI networks of normal breast cells (grade 0), well-differentiated cells (grade 1), moderately differentiated cells (grade 2), and poorly differentiated cells (grade 3)**. **(A)** A complex of chemokine proteins. **(B)** The Human Leukocyte Antigen (HLA) system. Nodes and edges are colored according to the corresponding network. Edge widths are proportional to the strength of interaction. Node dimensions are proportional to the gene expressions. Solid lines (intra-edges) connect the nodes of the same network, while dashed lines (inter-edges) connect the aligned nodes.

The alignments can be visualized in 2D (Figure 11), by selecting them from the list of local alignments and clicking on the “Show alignment network” button (Figure 9). Aligned nodes are colored according to the network they belong to and their sizes are proportional to the genes expressions. Edges are divided into two categories: intra-edges and inter-edges. Intra-edges connect nodes of the same subnetwork and are represented with solid lines with variable width, depending on the interaction weights. Inter-edges connect aligned nodes of different networks and are drawn with dashed black lines. In both cases, we used the Constraint-Based Layout (COLA) for network visualization.

### 3.5 Case study

In this section, we show a practical usage of SPECTRA through a case study. We compared a set of four TS-PPI networks, built from genes expression data in normal and well differentiated, moderately differentiated, and poorly differentiated breast cancer tissues. The aim is to identify subnetworks of differentially expressed genes across the normal breast and the three different grades of breast tumors.

#### 3.5.1 Data Preprocessing

We downloaded four breast cancer expression datasets for which information about the stage of breast tumors were available: GSE2361 (Ge et al., 2005), GSE2990 (Sotiriou et al., 2006), GSE4922 (Ivshina et al., 2006), and GSE7390 (Desmedt et al., 2007). We normalized data using RMA in R Bioconductor package (McCall et al., 2010).

The four expression datasets were then combined using COMBAT (Johnson and Li, 2007) into the R InSilicoDbMerging package. Finally, we grouped samples of the integrated dataset into four categories according to the grade of breast tumor (0 for normal tissue, 1 for well-differentiated tumor cells, 2 for moderately differentiated cells, and 3 for poorly differentiated cells). For each category, we computed the average expression value of each gene among samples. Results are stored into four different files (one per category).

#### 3.5.2 Uploading Data in SPECTRA and Building Breast TS-PPI Networks

We loaded the expression files in the “Expression data” panel in SPECTRA (Figure 5B) and we selected BioGRID and IntAct as PPI datasets in the “Interaction data” panel (Figure 5C). SPECTRA builds four TS-PPI networks, each of them has 7,472 nodes and 29,765 edges. We added each network to the comparison list of GASOLINE (Figure 8), by clicking on *Add to compare list* from the Result panel (Figure 6).

#### 3.5.3 Results of GASOLINE on TS-PPI Networks

Networks have been aligned by clicking on *Run GASOLINE* with the following parameters:

- Sigma = 1;
- Alpha = 0.05;
- Overlap threshold = 0.5;
- Refine iterations = 10;
- Minimum complex size = 2;
- Maximum gene expression log fold change threshold = 0.3;
- Use gene names for homology score.

GASOLINE took 27 s to complete the task and returned 20 local alignments. In Figure 11, the two biggest alignments are shown using the SPECTRA visualization tool. Both alignments contain genes that are known to be involved in breast cancer at different stages.

More precisely, the major group of aligned nodes in Figure 11A is formed by the chemokine proteins (CXCL10, CXCL9, CXCL11, CCL5) and the chemokine receptors CXCR3 and CCR1, which are all highly overexpressed across the different grades of breast tumor. Chemokines can be responsible for leukocyte migration during processes of tissue development and formation, or can attract immune cells to a site of inflammation. Chemokines and chemokine receptors are known to have an important role on cancer metastasis, by facilitating tumor dissemination (Muller et al., 2000; Karnoub and Weinberg, 2007). DPP4 gene has a lower expression variation but ensures the communication between CCL5, CCR1, and the other chemokine proteins. This result agrees with the key role of DPP4 in signal transduction and tumor progression (Pro and Dang, 2004).

The alignment of Figure 11B is characterized by the Human Leukocyte Antigen (HLA) system (HLA-DRB1, HLA-DMB, HLA-DMA, HLA-DRA). The HLA system is composed by proteins on cell surface that are responsible for regulation of the immune system. HLA genes exhibit very high differential expression between normal and tumor cells and their overexpression in breast cancers is confirmed by several papers (Bartek et al., 1987; Kaneko et al., 2011; Da Silva et al., 2013).

The above case study highlights the capability of SPECTRA in helping researchers in producing novel biologically sound hypothesis and insight in the study of tissue-specific diseases.

## 4 Discussion

SPECTRA is a knowledge base to build and compare tissue or tumor-specific PPI networks. It overcomes the current PPI network analysis limitations mainly due (i) to the spreading of data in several databases with low overlap; (ii) to be unaware of the role of proteins in human tissues and diseases. SPECTRA integrates 13 databases of both protein–protein interactions and expressions data. Moreover, it provides an algorithm to compare built-in or custom tissue and tumor-specific PPI networks and identify subnetworks of differentially expressed genes. Finally, the results can easily browsed trough a lightweight web application equipped with a 2D visualization network tool based on Cytoscape.js. Experiments performed on four TS-PPI networks built from gene expression data consisting of normal and breast cancer tissues show that the comparison algorithm can produce biologically significant results. SPECTRA database will go under update twice a year, with a semi-automatic curation of data downloaded from the online repositories. Future developments of SPECTRA aim to provide further network mining algorithms devoted to the analysis of expression data and the validation and annotation with ontologies of results.

## Conflict of Interest Statement

The authors declare that the research was conducted in the absence of any commercial or financial relationships that could be construed as a potential conflict of interest.

## Footnotes

^1^http://cancergenome.nih.gov

^2^http://thebiogrid.org

^3^http://dip.doe-mbi.ucla.edu/dip

^4^http://www.hprd.org

^5^http://www.ebi.ac.uk/intact

^6^http://mint.bio.uniroma2.it/mint

^7^http://www.ebi.ac.uk/arrayexpress

^8^http://www.ncbi.nlm.nih.gov/geo

^9^http://www.proteinatlas.org

^10^http://js.cytoscape.org
